# Southern Dental Association

**Published:** 1869-05

**Authors:** W. H. Morgan

**Affiliations:** Nashville, Tenn.


*Southern Dental Association.*—In the March No. of the *Journal* we briefly noticed the unjust charges made against us by J. H. McQ—in the *Dental Cosmos,* for our efforts to enlist the co-operation of Southern Dentists in the formation of a "Southern Dental Association."

Judging it best for others, who have been longer identified with the "American Dental Association," and more conversant with its proceedings, to notice at length the criticisms of J. H. McQ. our answer was confined to a mere defence against the charge of improper motives in advocating the formation of another association.

Dr. W. H. Morgan of Nashville Tennessee, who has for a number of years been one of the most prominent members of the American Dental Association, and who is thoroughly conversant with its private as well as public proceedings, replies to J. H. McQ. in the Feb. No. of the *Dental Register*, and brings forward such an array of facts as to prove conclusively that the statements we first published in the Journal were altogether correct.

We trust this reply of Dr. Morgan may be read by every Southern Dentist, and in order that it may reach all our readers we publish it in full;

Mr. Editor: In the *Cosmos* for January, 1869, under the above caption, there are some criticisms that deserve a passing notice, on account of their manifest unfairness and perversion of facts. J. H. McQ. quotes: "The impression has become general among Southern Dentists that sectional feelings govern the action of the majority of the American Dental Association." True, sir. And then says: "It would be unjust, however, to permit such unfounded statements to pass unanswered. To refute this it is only necessary to turn to the elections of officers for the past few years," and goes on to enumerate five gentlemen who have been elected to office from the Southern, or late "slave-holding States." "The evidence," he says, "thus presented of the absence of sectional feelings is overwhelming, for, although the attendance on the part of Southern practitioners has been limited for other reasons than those that are given above. The proposition of officers each year has been *decidedly* in their favor," and quotes what he said at Chicago as a "settler to this question." "We have come hundreds of miles away from home, let each and all, therefore, turn out the silver lining of their manhood,etc." Let us examine as to the officers. This Association has had eight elections of officers, forty-eight in all, and up to this date two Southern, just two Southern born men, (the writer and Dr. Rodgers, of Kentucky) and no more have been elected to office in the Association. The others whom he names as being Southern men are from the North, and most of them are what, in common parlance, are termed "Yankees," and have simply "emigrated South." Some of them have boasted to the writer that they were live Yankees. So two in forty-eight is decidedly in their favor. This has not been the result of accident, but of preconcerted action on the part of Northern members, so he says, (who doubts it ?) but not for the reasons assigned. This Association was organized in Washington City (Southern soil) and has never met in a Southern State since that date,(1860) although frequently invited and urged by Southern delegates to do so. Why ? Because it has been controlled by the "action of a majority of its members," who are Northern men, and for reasons satisfactory to themselves the place of meeting has been fixed in some of the Northern States. At the meeting in Chicago, when J. H. McQ. so eloquently exhorted his brethern to "to turn out the silver lining of their manhood," and while the name of the writer was before the nominating committee, he was approached by a member of that committee direct from the committee, and his politics asked. It was not doubted that the committee sent him. And again immediately after the election of officers another member of that committee approaching him, said, in an apologetic tone and manner, "I voted for you, and you would have been elected but for some doubts as to your loyalty, but you know, sir, this is a National affair, and it would not do to elect any one to an office in it but Union men."

The next year at Boston, the writer in the chair, a motion was put and carried to invite General Butler to visit the Association. When the nays were called for some one voted nay rather loudly, when a Northern brother, in a rather boisterous tone said, "Some of us are abolitionists, let him come in." The offensive manner in which this was done was unmistakable. After General B. retired from the hall, a resolution was offered and passed, "That this Association is happy to see and hear Major General Butler," etc. (See proceedings, p. 241, with protest, which reads, "The undersigned members and delegates to the American Dental

Association earnestly protest against the action of the majority of this body in refusing to reconsider the vote by which the above resolution was passed. The ground of our protest is this: The subject matter contained in said resolution we regard as wholly foreign to the objects for which this body was organized.") This protest was signed by ten delegates. While the motion to reconsider was pending, J. H. McQ. "turned out the silver lining of his manhood," by making a speech against it, and thereby defeated it by a small majority. A manly, generous appeal was made in its favor by L. D. Sheperd, of Boston, Massachusetts, and an entreating one by F. Y. Clark, of Savannah, Georgia (he who boasts the secret service medals given to him by General Wilson and others of the U. S. Army.), but no appeals would avail. The resolution was spread upon the minutes and published. And yet J. H. McQ. says: "And those who have attended the meetings from that section (South) and participated in the proceedings, will acknowledge that other than sectional or political questions have *fully* engaged the time and attention of the delegates." In the face of the above facts 'they will make no such acknowledgment. This, Mr. Editor, is the manner in which "year after year the olive branch has been cordially extended by the Northern members of the Association to their professional brethern in the South.

Without inquiring into the motives of one making such erroneous statements as quoted, we place these facts before our profession as some of the reasons why a Southern Dental Association is desired. The author of the article in the *American Journal of Dental Science*, which has so offended J. H. McQ., is not known to the writer and he has no interest in defending him. This article has been written solely in the interest of truth, and in no offensive sense, whatever. The writer does not mean to be offensive to any one alluded to, and has no unkind feeling to any member of the Association. He has attended five meetings of the Association, has been honored by it with office, and personally has always been treated with the utmost courtesy and consideration by his Northern brethern. He hopes to be on hand again in due time.

Nashville, Tenn. W. H. Morgan, M.D.,D.D.S.

